# Subacute Thyroiditis After Receiving the Adenovirus-Vectored Vaccine for Coronavirus Disease (COVID-19)

**DOI:** 10.7759/cureus.16045

**Published:** 2021-06-29

**Authors:** Samson O Oyibo

**Affiliations:** 1 Diabetes and Endocrinology, Peterborough City Hospital, Peterborough, GBR

**Keywords:** subacute thyroiditis, coronavirus vaccine, covid 19, adenovirus-vector, overt hypothyroidism

## Abstract

Since the emergence of the coronavirus disease pandemic, several effective vaccines have been introduced. These vaccines work through several different immunogenic pathways to produce effective immunity. There have been a number of reports of patients developing subacute thyroiditis and thyroid dysfunction after receiving the coronavirus (COVID-19) vaccine. This paper presents a case of a female patient who developed subacute thyroiditis soon after receiving the adenovirus-vectored COVID-19 vaccine. The patient presented with severe neck pain and her blood results demonstrated an initial thyrotoxic phase followed by a hypothyroid phase. She had no past history of thyroiditis or thyroid dysfunction. Subacute thyroiditis occurring after COVID-19 vaccination is rare but also probably underreported. We hope that this case report not only contributes to the literature but also raises awareness of subacute thyroiditis occurring after receiving the COVID-19 vaccine.

## Introduction

The emergence of severe acute respiratory distress syndrome secondary to coronavirus infection (SARS-CoV-2, COVID-19) in late 2019 posed a significant threat to the entire world. Since then several effective vaccines have been introduced. Different vaccines produce immunity through different mechanisms. Some vaccines contain adjuvants, which are mainly used to increase the response to vaccination in the population. However, adjuvants can induce other autoimmune and inflammatory reactions by stimulating immunogenic cross-reactivity in genetically susceptible individuals [1]. Several cases of subacute thyroiditis have been reported after exposure to other vaccines [2-5], but there are not many reports related to exposure to the COVID-19 vaccine. This paper presents a case of a patient who developed subacute thyroiditis soon after receiving the adenovirus-vectored COVID-19 vaccine (Oxford-AstraZeneca, United Kingdom). The objective of the article is to create awareness regarding the possible association between receiving the COVID-19 vaccine and the onset of subacute thyroid dysfunction. This incident was appropriately reported to the Medicines and Healthcare products Regulatory Agency (MHRA), United Kingdom.

## Case presentation

Medical history and demographics

A 55-year-old woman presented with neck pain and swelling, which had been going on for four weeks. This was accompanied by headache, sore throat, generalized aches and palpitations. She had received her first dose of the COVID-19 vaccine (ChAdOx1 nCoV-19 vaccine, AstraZeneca) three weeks prior to the onset of these symptoms. There was no history of any viral or respiratory illness prior to the onset of symptoms. Her past medical history consisted of well-controlled asthma. She had no family history of hypothyroidism or thyroiditis. She was a non-smoker and worked as community healthcare professional. On initial examination, she had a very tender goiter with no other abnormal clinical findings.

Investigations

**Figure 1:**
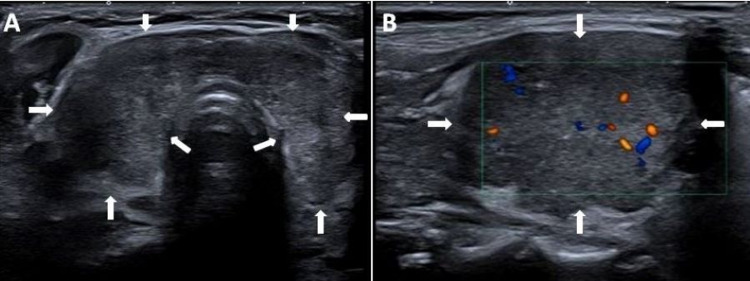
Ultrasound scan of the thyroid gland Enlarged thyroid gland with heterogeneous echotexture (A), and Doppler studies of right thyroid lobe showing reduced vascular flow (B).

Initial blood results revealed raised inflammatory markers and thyroid hormone levels, consistent with the thyrotoxic phase of subacute thyroiditis. Her thyroid peroxidase antibody (anti-TPO) test was negative. Other routine blood results were normal (Table 1). A thyroid ultrasound scan demonstrated an enlarged thyroid gland with heterogeneous echotexture throughout (Figure 1). There were no nodules and no hyper-vascularity. Features were in keeping with thyroiditis.

**Table 1:** Initial blood tests and results demonstrating raised inflammatory markers and raised thyroid hormone levels consistent with subacute thyroiditis

Blood test	Result	Reference range
Sodium (mmol/L)	139	132-145
Potassium (mmol/L)	4.5	3.4-5.1
Chloride (mmol/L)	105	97-110
Creatinine (mmol/L)	53	45-84
Urea (mmol/L)	5.0	2.5-7.8
Thyroid-stimulating hormone (mU/L)	0.09	0.3-4.2
Free thyroxine (pmol/L)	25.2	12.0-22.0
Total protein (g/L)	71	60-80
Albumin (g/L)	44	35-50
Globulin (g/L)	27	20-35
C-reactive protein (mg/L)	87	<5
Hemoglobin (g/L)	119	115-165
White cell count (10^9^/L)	8.5	4.0-11.0
Platelet count (10^9^/L)	491	150-400
Erythrocyte sedimentation rate (mm/hr)	51	0-18
Anti-thyroid peroxidase antibody (IU/ml)	<10	<34

Treatment

The patient was initially treated with a beta-blocker (propranolol) to control the palpitations. For the neck pain, she used over-the-counter ibuprofen and paracetamol.

Outcome and follow up

A repeat thyroid function test performed after six weeks demonstrated results in keeping with severe hypothyroidism (Table 2) and she was commenced on levothyroxine 50 micrograms daily. A repeat test performed six weeks later demonstrated improvement. Her thyroid receptor antibody (TRAb) was negative, but her thyroglobulin antibody test was positive. Her levothyroxine dose was increased to 75 micrograms daily and she remains well.

**Table 2:** Follow-up blood test results at six and 12 weeks demonstrating severe hypothyroidism at six weeks, improvement while taking levothyroxine at 12 weeks and raised thyroglobulin antibody level at 12 weeks

Blood test	Reference range	Results at 6 weeks	Results at 12 weeks
Thyroid-stimulating hormone (mU/L)	0.3-4.2	20.3	5.35
Free thyroxine (pmol/L)	12.0-22.0	4.7	15.6
Free triiodothyronine (pmol/L)	3.1-6.8	-	4.2
Thyroid receptor antibody (IU/L)	<2.9	-	1.9
Thyroglobulin antibody (kU/L)	<3.0	-	15

## Discussion

COVID-19 is an infectious disease caused by the novel coronavirus, SARS-CoV-2. This virus was first identified in Wuhan, a city in the Hubei Province of China [6]. Global efforts were directed towards the development of a vaccine against COVID-19. There are now several vaccines being used with positive effects on morbidity and mortality [7,8]. The common side effects of these vaccines, such as pain in the injected site, allergic skin reactions, flu-like symptoms, headache, fatigue and fever, have been well documented [9,10]. A large study revealed that the systemic and local side-effects from receiving COVID-19 vaccination occurred at frequencies lower than reported in the phase-3 trials [11].

Subacute thyroiditis is a spontaneously remitting inflammatory condition of the thyroid gland that can last for weeks to several months. Females account for 75%-80% of cases. Viral infections, immune-modulatory drugs and postpartum autoimmunity have been implicated as etiological factors. Patients characteristically present with severe pain, swelling and tenderness in the thyroid region, accompanied by malaise, fatigue, myalgia and arthralgia. There is usually an initial thyrotoxic phase with raised inflammatory markers, followed by a hypothyroid phase and then a recovery phase. Symptoms are generally self-limiting. However, the neck pain can be severe and persistent, requiring a short course of steroid (prednisone) therapy [12].

Cases of subacute thyroiditis occurring after viral vaccines have been reported [2-5]. Following the COVID-19 pandemic, there have been reports of thyroid dysfunction occurring after the COVID-19 vaccination. A recent report involved a 42-year-old female who developed subacute thyroiditis within a week of receiving the mRNA COVID-19 vaccine (SARS-CoV-2 vaccine, Pfizer-BioNTech) [13]. Another report involves three females who developed subacute thyroiditis soon after receiving the inactivated coronavirus vaccine (SARS-CoV-2 vaccine, Sinovac Biotech Ltd) [14]. A more recent report involves two females who developed Graves’ disease with thyrotoxicosis soon after receiving the mRNA COVID-19 vaccine [15]. The mechanism for post-vaccination subacute thyroiditis or thyroid dysfunction remains unknown. However, adjuvants contained in vaccines (used mainly to increase the response to vaccination in the general population) may play a role in producing diverse autoimmune and inflammatory responses [15,16]. The potential for cross-reactivity between the coronavirus spike protein target produced by the mRNA vaccine and healthy thyroid cells antigens has also been mentioned [17]. The possible interactions between genetic predisposition, a history of other autoimmune conditions, the presence of adjuvants, and cross-reactivity between spike proteins and healthy thyroid cell antigens, all require further investigation.

Subacute thyroiditis occurring after COVID-19 vaccination is rare and probably underreported. Our case may be one of the very few reports of a woman developing subacute thyroiditis after receiving the adenovirus-vectored COVID-19 vaccine. Although viral infection is a more common cause of subacute viral infection, our patient did not have any preceding viral or respiratory symptoms before developing severe neck pain and swelling. Her symptoms occurred three weeks after receiving the vaccine.

## Conclusions

Subacute thyroiditis occurring after receiving the COVID-19 vaccine is a rare and probably underreported phenomenon that seems to have a predilection for the female gender. Further investigation is required to evaluate the possible predisposing factors to developing subacute thyroiditis after receiving the COVID-19 vaccine. We hope that this case report not only contributes to the literature but also raises awareness of subacute thyroiditis occurring after receiving the COVID-19 vaccine.
